# Capturing cognitive efficacy in Alzheimer's disease clinical trials. A review of the last 25 years.

**DOI:** 10.1002/Alz70859_100004

**Published:** 2025-12-25

**Authors:** John E Harrison

**Affiliations:** ^1^ Alzheimer Center Amsterdam, Neurology, Vrije Universiteit Amsterdam, Amsterdam UMC location VUmc, Amsterdam, Amsterdam, Netherlands; King's College ‐ Institute of Psychiatry, Psychology & Neuroscience, London, London, United Kingdom; Metis Cognition Ltd., Kilmington, Wiltshire United Kingdom

## Abstract

Many tests have been used to study memory in Alzheimer’s disease (AD) and related dementias. At the turn of the century, these assessments included the CANTAB system, MMSE, Cognitive Drug Research (CDR), RBANS, and Cogstate, along with a large set of established paper and pencil tests. Several additional assessments have been developed and used in observational studies and clinical trials since 2000, including the mini‐cog (2000) and MOCA (2005) as well as the NIH toolbox (2004). However, since the registration of the first AD drugs, the preferred cognitive assessment has been the ADAS‐cog. This is despite several substantial criticisms levelled at this test and despite expanding options in cognitive assessments.

The ADAS‐cog has issues with ceiling effects, lack of parallel forms of the praxis and language tests and generally poor content validity, and the attempt to add items to the ADAS‐cog to solve these issues has not been successful. The persistent use of the ADAS‐cog seems unaffected, despite the fact that better tests of some missing domains, such as verbal fluency, coding, attention and working memory have all demonstrated assay sensitivity in early AD.

The lack of assay sensitivity of the ADAS‐cog in mild AD (MMSE 21 to 26) in the phase 2 AN1792 study, presented side‐by‐side with the newly proposed NTB showing comparable mild and moderate sensitivity, inspired development of several optimized cognitive composites for early AD stages from 2010‐2020. These included the PACC to separate amyloid positive and negative individuals, and the APCC, API‐LOAD and PACC‐5 for measuring progression in the pre‐MCI stage. Also in the late 2010s, additional composite outcomes were proposed that included both cognition and global or functional scale items, and were intended primarily for the early AD stage of disease (ADCOMS, iADRS).

While the ADAS‐Cog has aided in the search for effective AD treatments, a reflection on the past 25 years of the development of cognitive assessments reveals that much effort has been spent to overcome its deficiencies and the AD world would experience more rapid advancement if we make better use of the tools we have and continue to address deficiencies.

